# Interventions promoting healthy eating as a tool for reducing social inequalities in diet in low- and middle-income countries: a systematic review

**DOI:** 10.1186/s12939-016-0489-3

**Published:** 2016-12-22

**Authors:** Ana-Lucia Mayén, Carlos de Mestral, Gerardo Zamora, Fred Paccaud, Pedro Marques-Vidal, Pascal Bovet, Silvia Stringhini

**Affiliations:** 1 Institute of Social and Preventive Medicine (IUMSP), Lausanne University Hospital, Bâtiment Biopôle 2, Route de la Corniche 10, 1010 Lausanne, Switzerland; 2 Evidence and Programme Guidance, Department of Nutrition for Health and Development, World Health Organization, Geneva, Switzerland; 3 Department of Internal Medicine, Lausanne University Hospital, Lausanne, Switzerland

**Keywords:** Interventions, Education, Inequalities, Diet, Low- and middle-income countries

## Abstract

**Introduction:**

Diet is a major risk factor for non-communicable diseases (NCDs) and is also strongly patterned by socioeconomic factors. Whether interventions promoting healthy eating reduce social inequalities in diet in low- and middle-income countries (LMICs) remains uncertain. This paper aims to summarize current evidence on interventions promoting healthy eating in LMICs, and to establish whether they reduce social inequalities in diet.

**Methods:**

Systematic review of cross-sectional or quasi-experimental studies (pre- and post-assessment of interventions) in Pubmed, Scielo and Google Scholar databases, including adults in LMICs, assessing at least one outcome of healthy eating and showing results stratified by socioeconomic status.

**Results:**

Seven intervention studies including healthy eating promotion, conducted in seven LMICs (Brazil, Chile, Colombia, Iran, Panama, Trinidad and Tobago, and Tunisia), met our inclusion criteria. To promote healthy eating, all interventions used nutrition education and three of them combined nutrition education with improved acces to foods or social support. Interventions targeted mostly women and varied widely regarding communication tools and duration of the nutrition education sessions. Most interventions used printed material, media use or face-to-face training and lasted from 6 weeks to 5 years. Four interventions targeted disadvantaged populations, and three targeted the entire population. In three out of four interventions targeting disadvantaged populations, healthy eating outcomes were improved suggesting they were likely to reduce social inequalities in diet. All interventions directed to the entire population showed improved healthy eating outcomes in all social strata, and were considered as having no impact on social inequalities in diet.

**Conclusion:**

In LMICs, agentic interventions promoting healthy eating reduced social inequalities in diet when specifically targeting disadvantaged populations. Further research should assess the impact on social inequalities in diet of a combination of agentic and structural approaches in interventions promoting healthy eating.

**Electronic supplementary material:**

The online version of this article (doi:10.1186/s12939-016-0489-3) contains supplementary material, which is available to authorized users.

## Introduction

Diet is a major risk factor for non-communicable diseases (NCDs) [1]. The social patterning of diet, or how dietary habits vary by social group, may partly explain social differences in the burden of NCDs, whereby those with unhealthier diets are more likely to have higher NCD rates. In high-income countries (HICs), individuals with low socioeconomic status (SES) tend to have a higher intake of fat, salt and sugar [2–4] while in many low- and middle-income countries (LMICs) the opposite trend is observed, i.e. individuals with high SES tend to first adopt unhealthy diets rich in added sugars and fat [5, 6]. However, the social patterning of diet in LMICs is expected to increasingly resemble that observed in HICs along with socioeconomic development. According to this framework, in the near future the most disadvantaged groups in LMICs will adopt unhealthy dietary patterns [7, 8] and will experience higher NCD rates than their most advantaged counterparts [7], a pattern that is already observed in several middle income countries [8, 9].

The global prevalence of NCDs is rapidly increasing, especially in LMICs, where almost 75% of NCD-related deaths took place in 2012 [10]. In 2011, the General Assembly of the United Nations adopted a political declaration agreeing on approaches for the prevention and control of NCDs [11], taking into account the social determinants of health (SDH) [12]. Further in 2013, the World Health Organization launched the Global Action Plan for the prevention and control of NCDs for the 2013–2020 period prioritizing the SDH [10]. For this purpose, different approaches have been suggested: to focus on disadvantaged populations, on the entire population, or on a combination of both [13–16].

In many HICs, preventive efforts to reduce social inequalities in diet have, for instance, taken the form of structural or agentic interventions. Structural interventions work by altering the context in which health is produced or reproduced [17], such as taxes on unhealthy foods or subsidies on fruits and vegetables [18] and reformulation of food products (i.e. lowering the salt content) [19]. Agentic interventions are the ones in which an individual must act on information provided, such as health education programs [20–22]. Such interventions have been successful in reducing social inequalities in diet in several HICs [20–23]. In LMICs, several strategies have been implemented to promote healthy eating, such as efforts to increase fruit and vegetable intake through school gardens and local production, or interventions to decrease salt and fat intake through food reformulation and labeling [24]. Overall, interventions to promote healthy eating have frequently included nutrition education on the consequences of dietary behaviors [25–27], and empower consumers to act on such information by changing their attitudes and behaviors towards food [13, 28]. However, most interventions promoting healthy eating in LMICs do not specifically aim at reducing social inequalities in diet, and their effect on the social patterning of diet remains unknown.

The objective of this review is to summarize current evidence on interventions promoting healthy eating in LMICs, and to establish whether they reduce social inequalities in diet.

## Methods

### Search strategy

Following the PRISMA guidelines, studies were identified by searching PubMed, Scielo and Google Scholar electronic databases. No restrictions were set for year of publication. The last search was run on the 8^th^ of March 2016. The free search terms included in the search are summarized in Additional file 1: Table S1. Additionally, we included the following MeSH terms in Pubmed: Chronic Disease, Diabetes Mellitus, Cardiovascular Diseases, Obesity, Hypertension, Coronary Disease, Cerebrovascular Disorders, Neoplasms, Respiration Disorders, Chronic Disease/diet therapy, Nutrition Policy, Nutrition Therapy, Diet Therapy, Developing Countries, Asia, Africa, Latin America, South America and soecioeconomic factors. Reference lists of included studies were also searched to identify potential articles.

Exclusion criteria were: 1) pharmacological interventions; 2) prevalence or qualitative studies; 3) studies conducted on HICs; 4) studies conducted on undernourished or diseased individuals, children, adolescents or non-representative populations (e.g. studies in prisioners or caregivers); 5) articles written in languages other than English, French, Spanish or Portuguese, and 6) non-peer-reviewed studies, duplicate publications or articles restricted to an abstract. Interventions conducted in children and adolescents were excluded as the target population were adults and dietary practices differ according to age [29]. Studies including individuals with a body mass index ≥ 25 kg/m^2^ were included as such participants were not considered as diseased.

Inclusion criteria were: 1) studies conducted in LMICs, defined according to the World Bank classification [30]; 2) cross sectional or quasi-experimental, peer-reviewed studies; 3) focusing on interventions promoting healthy eating among adults (≥18 years) or households; 4) measuring at least one healthy eating outcome [20] (i.e. fruit and vegetable intake or changes in food behavior) and 5) showing results stratified by at least one SES indicator (i.e. household assets, education, income, occupational position or area-based SES indicators).

For all articles initially selected, the title and abstract were separately assessed by two reviewers for eligibility (ALM and COVD). Disagreements were resolved by discussion. Data extraction for each study included country where the study was conducted, period of intervention, sample size, target population, SES categories, communication tools, and duration of the nutrition education sessions. Total duration of interventions, individual health behavior change model used, expected outcome measures and effects of healthy eating were also exctracted.

All interventions were categorized according to two criteria: 1) those focused on disadvantaged populations categorized as such by each included study; or 2) those directed to the entire population but stratifying results by SES. In this study, social inequalities in diet refer specifically to differences in dietary intake between socioeconomic groups, not by other social stratifiers such as age, sex or ethnicity. The effect of each intervention on social inequalities in diet was assessed using a similar approach to the one used in a previous study [20]. Interventions were defined as: 1) likely to reduce social inequalities if healthy eating outcomes had improved more in low vs. high SES individuals or households (e.g. more servings per day of fruit and vegetable consumption in low vs. high SES groups); 2) likely to increase social inequalities if healthy eating outcomes had improved more in the high vs. low SES individuals or households (e.g. more servings per day of fruit and vegetable consumption in high vs. low SES groups), and 3) likely to have no impact on social inequalities if healthy eating outcomes reduced or increased similarly in individuals and households of all SES groups.

## Results

### Included studies

The study selection procedure is shown in Additional file 2: Figure S1. The search of the three databases plus hand searched articles provided a total of 249 studies. Most excluded studies were conducted in children, adolescents or diseased individuals, assessed no intervention, or did not stratify results by SES. A total of seven articles were included; their main characteristics are shown in Table 1. All studies used nutrition education to promote healthy eating. Studies assessed interventions conducted between 2002 and 2014 in seven LMICs: five in the Americas and the Caribbean (Brazil [31], Chile [32], Colombia [33], Panama, Trinidad and Tobago [34]), and two in the Middle East and Africa (Iran [35], Tunisia [36, 37]). One study focused on families [31], one on private sector employees [37], and four on women [32, 34, 35, 38]. One study included individuals with risk factors (body mass index ≥ 25 kg/m^2^) [32].

**Table 1:** Main characteristics of the studies included in the present review

Study	Country	Setting	Period of intervention	Sample size	Target population	SES categories
Interventions focused on disadvantaged populations						
Constante Jaime P, et al. 2006 [31]	Brazil	Community in Sao Paulo	2006	36 households	Households in low SES district (Grajaú)	Number of household assets Schooling of household member responsible for purchasing and preparing food
Lucumi DI, et al. 2006 [33]	Colombia	Neighborhoods in Bogota	2006	97 women	Women in low SES neighborhoods	Low SES neighborhood participating in social programs
White SC, et al. 2006 [34]	Panama, Trinidad and Tobago	Church groups, public clinics, community organizations	2006	100 women	Low SES women	SES
Vio F, et al. 2011 [32]	Chile	Health centers in Peñalolén community	2011	480 women	Low SES women	Low SES: monthly household income <510 USD
Interventions addressing the entire population						
Zammit N, et al. 2015 [36]	Tunisia	Clinical settings	2010–2013	Adults in 1000 households (1880 pre-intervention; 1977 post-intervention)	Communities from 16 districts	Low & middle SES/middle & high SES
Bhiri S, et al. 2015 [37]	Tunisia	Workplaces	2009–2014	3888 employees (1775 pre-assessment; 2113 post-assessment)	Governorate of Sousse	Low & middle SES employees, schooling and job position
Sadeghi M, et al. 2011 [35]	Iran	Media, books, health centers and literacy centers	2002–2007	10,586 women (6105 pre-intervention; 4481 post-intervention)	Working women and homemakers in three counties (Isfahan, Arak and Najafabad)	Job position

*SES* socioeconomic status, *USD* American dollars

### Description of interventions promoting healthy eating

All included interventions are summarized in Table 2. Four of the seven interventions focused on disadvantaged populations only [31, 32, 34, 35, 38]. Two used printed material for education purposes [32, 38] and one used media and face to face training [34]. Their total duration ranged from 6 weeks in Panama and Trinidad and Tobago [34] to 6 months in Chile [32]. Two of the four interventions used a behavior change model to plan the nutrition education sessions [34, 38]. One intervention was a healthy eating intervention only [31] while the others aimed at improving healthy eating plus increasing physical activity [32, 34, 38], decreasing smoking [38] or increasing cancer screening [34].

**Table 2:** Description of interventions promoting healthy eating to reduce social inequalities in diet

Study	Communication tools	Duration of nutrition education sessions	Total duration of intervention	Individual health behavior change model used	Expected outcome
Interventions focused on disadvantaged populations					
Constante Jaime P, et al. 2006 [31]	─	Three meeting of 2 h each	5 months	─	Increase FV intake
Lucumi DI, et al. 2006 [33]	Printed material	Weekly sessions of 2 h each	4 months	Social cognitive theory	Increased FV intake Forbidding in-home smoking Increased PA
White SC, et al. 2006 [34]	Video presentation, face to face training	Weekly meetings of unknown duration	6 weeks	Theory of implementation intentions and social support	Increased FV intake Increased PA Increase participation in cancer screening
Vio F, et al. 2011 [32]	Printed material	Three workshops of unknown duration	6 months	─	Food behavior change Increased PA
Interventions addressing the entire population					
Zammit N, et al. 2015 [36]	Printed material, radio	─	3 years	─	Increased FV intake Increased PA Decrease of tobacco use
Bhiri S, et al. 2015 [37]	Face to face training, printed material, tv	─	3 years	─	Increased FV intake Increased PA Decrease of tobacco use
Sadeghi M, et al. 2011 [35]	Face to face training, printed material, radio/tv	From 5 min to 2 h every week	5 years	─	Food behavior change Increased PA Decrease of tobacco use

*FV* fruit and vegetables, *PA* physical activity

Three interventions were directed to the entire population [35–37]. All used printed material and media for nutrition education purposes, and two used face to face training [35, 37]. Their total duration ranged from 3 to 5 years and aimed at improving healthy eating, physical activity and decreasing tobacco use.

### Healthy eating outcomes and reduction of social inequalities in diet

The effect of interventions promoting healthy eating and their impact on social inequalities are described in Table 3. Two of the four interventions focusing on disadvantaged populations reported an increase in fruit and vegetable consumption after the intervention [31, 38], engendering a decrease in the overall inequalities in diet while assuming that all conditions were kept equal for the advantaged group. One study reported a significant decrease in fruit and vegetable intake after the intervention [34], being likely to increase social inequalities in diet. Another study reported food behavior change after the intervention (i.e. increased intake of skim milk and whole bread), being likely to reduce social inequalities in the intake of these specific food items [32].

**Table 3:** Healthy eating outcomes and effect of healthy eating interventions to reduce social inequalities in diet

		Healthy eating outcomes		
Study	Measures for outcomes	Effect	No effect	Effect on social inequalities in diet
Interventions focused on disadvantaged populations				
Constante Jaime P, et al. 2006 [31]	% FV intake in respect to total food energy purchased before and after the intervention	The % increased 2.58%	─	↓
Lucumi DI, et al. 2006 [33]	Intake of FV	Vegetables or salad intake increased from 44 to 65%	Fruit intake increased from 55 to 56% (*p* = 0.87)	↓
White SC, et al. 2006 [34]	Intake of 4 or more servings of FV/day	FV intake of 4 or more servings/day decreased from 45 to 2% in Panama and from 45 to 19% in Trinidad and Tobago	─	↑
Vio F, et al. 2011 [32]	Changes in food behavior	Skim milk and whole bread intake increased from 0.2 day/week to 0.4 day/week and 0.6 day/week to 1.6 day/week respectively in intervention group	FV intake did not significantly increase after intervention	↓
Interventions addressing the entire population				
Zammit N, et al. 2015 [36]	Intake ≥5 servings of FV/day	FV intake increased from 29 to 43% in low & middle SES and from 40 to 61% in middle & high SES participants in intervention group	─	↔
		FV intake increased from 46 to 69% in the low & middle SES participants and from 56 to 70% in the middle & high SES participants in control group		
Bhiri S, et al. 2015 [37]	Intake ≥5 servings of FV/day	FV intake increased from 42 to 55% in intervention group of medium SES employees, and from 45 to 55% in intervention group of office staff employees	FV intake increased from 49 to 51% in intervention group of low SES employees (*p* = 0.43), and from 48 to 51% in intervention group of worker employees (*p* = 0.20)	↔
Sadeghi M, et al. 2011 [35]	Changes in food behavior (dietary index)	Global dietary index decreased from 1.03 ± 0.28 to 0.80 ± 0.30 in homemakers and from 1.12 ± 0.26 to 0.82 ± 0.32 in working women	─	↔

*FV* fruit and vegetables, *SES* socioeconomic status. The effect on social inequalities in diet is displayed symbolically in the table as: ↓ for an intervention likely to reduce social inequalities in diet (intervention improved dietary outcomes in individuals with low SES); ↑ for an intervention likely to increase social inequalities in diet (intervention improved dietary outcomes in individuals with high SES); ↔ for an intervention likely to have no impact on social inequalities in diet

Two of the three interventions focusing on the entire population showed a significant increase in fruit and vegetable intake in the entire population after intervention [36, 37], being likely to have no impact on social inequalities in diet. Another intervention showed a significant decrease of the global dietary index in the entire population after intervention (i.e. improved healthy eating) [35], also being likely to have no impact on social inequalities in diet.

## Discussion

To our knowledge, this is the first systematic review of the literature assessing interventions promoting healthy eating as a tool for reducing social inequalities in diet in LMICs. To improve healthy eating outcomes, all interventions used nutrition education and three of them combined nutrition education with improved access to foods or social support [31, 34, 38]. Most interventions (five out of seven) aimed to increase fruit and vegetable intake. Total duration of interventions did not seem to be related to the reduction of social inequalities in diet, as interventions which successfully reduced social inequalities in diet had the shortest duration (from 4 to 6 months). Interventions were focused either on disadvantaged populations or on the entire population. Three of the four interventions that focused on disadvantaged populations were likely to reduce social inequalities in diet (i.e. fruit and vegetables, skim milk and whole bread intake), and only one of these included an individual health behavior change model. None of the interventions directed to the entire population were likely to reduce social inequalities in diet.

### Description of interventions promoting healthy eating

Most interventions were focused on women only (four interventions), as they are usually in charge of food preparation and household food security [39]. However, further research is needed to assess the effectiveness of interventions directed to men in LMICs, who frequently hold the power of decision making and usually have a major role in the distribution of food portions in households. Also, evidence on the impact of healthy eating interventions by gender is scarce, which highlights the need for research on the differences between interventions on men and women’s dietary habits. Most interventions used printed material, including two of the three interventions which were likely to reduce social inequalities in diet. As both effective interventions targeted only disadvantaged populations, we cannot conclude on the effect of using equity-sensitive printed materials in structural interventions and further research is suggested.

The three interventions that were likely to reduce social inequalities in diet had the shortest duration (4 to 6 months), suggesting that duration is not related to the effect of an intervention on social inequalities in diet. This result has very important implications for prioritizing the allocation of resources in interventions [40]. Moreover, duration of interventions promoting healthy eating that successfully improved dietary patterns in HICs [41–43] and LMICs [44] varies considerably.

Only two of the seven interventions used an individual health behavior change model to plan the nutrition education sessions, and only one of these showed a reduction in social inequalities in diet. Thus, we cannot firmly conclude if the inclusion of behavior change models results in more effective interventions and greater benefit for participants [45].

### Healthy eating outcomes and reduction of social inequalities in diet

Evidence on interventions to reduce social inequalities in diet in LMICs remains scarce. In HICs, interventions to reduce health inequalities have been conducted for more than a century [15] and usually include structural or agentic approaches [16]. Structural approaches, which are those that promote health by altering the structural context in which health is produced [17], have usually targeted the entire population [46–49]. They may refer to nutrition-specific policies (i.e. policies aimed to influence food supply or consumption) or nutrition-sensitive policies (i.e. policies implemented outside of the health and food sectors). Some examples include food labeling, food reformulation, food taxes and subsidies, income and social protection policies [22]. Agentic approaches have usually targeted the entire population or disadvantaged populations only [50, 51] (e.g. nutrition education and health information campaigns) [22]. However, whether interventions should target only disadvantaged individuals, the entire population or combine both approaches is a matter of debate. Advocates of high-risk strategies highlight the need to direct resources towards individuals at high risk [52], while those in favor of population strategies argue that many people at a small risk may lead to more cases of disease than a few people with high risk, making these strategies temporary and palliative [53]. Thus, in HICs there is no “one-fits-all” approach. Several authors have proposed the two perspectives are compatible [15, 16] and should not be mutually exclusive but complementary [25] because focusing only on agentic interventions does not reduce the unequal population distribution of social and economic resources which cause dietary inequalities [54].

In LMICs, interventions to improve diet have mainly taken the form of nutrition education programs (i.e. agentic interventions) but most did not assess social inequalities in diet. Even though some studies assessed structural interventions (e.g. subsidies of fruit and vegetables), they could not be included in our review as results were not stratified by SES. Thus, our results are limited to agentic interventions. In our review, three interventions focusing on disadvantaged populations were likely to reduce social inequalities in diet. One educational intervention specifically targeting disadvantaged populations yielded the unexpected result of a decrease in fruit and vegetables consumption (although a causal effect of the intervention could not be proved). A qualitative study conducted by the authors showed that a likely explanation was the high price of fruit and vegetables and a preference for fried foods, carbohydrates and sweetened beverages among the participants. Several intervention trials in HICs have shown that nutrition education alone is insufficient to increase the intake of fruit and vegetables, but that the combination of nutrition education plus decreased prices is [22, 55]. This was the case in one of the interventions in our study which additionally included increased access to fruit and vegetables [31]. Thus, we were unable to disentangle the individual effects of the nutrition education sessions and accessibility to healthy foods on social inequalities in diet in LMICs. Similarly, two other studies in our review combined nutrition education sessions with social support [34, 38], and we could not disentangle their effects on social inequalities in diet. Further research is needed on interventions promoting healthy eating in LMICs, especially regarding increased purchasing power and social support to reduce social inequalities in diet.

The three interventions targeting the entire population improved healthy eating outcomes in all socioeconomic strata, thus being likely to have no impact on social inequalities in diet. These results are in line with evidence in HICs showing no impact on social inequalities in diet of interventions such as user-friendly food labeling (e.g. traffic light schemes), mandatory and standardized front-of-pack labeling, and population-wide control of unhealthy food marketing through mass media [22].

In our study, as all agentic interventions targeting the entire population failed to reduce social inequalities in diet, our results may suggest that structural interventions targeting the entire population can tackle the unequal distribution of factors that hinder the opportunity to eat a healthy diet [22]. One example in a HIC (New Zealand) is a targeted food pricing policy which was effective to improve the diet of the entire population and reduce dietary inequalities [56]. In LMICs, some examples of structural interventions to reduce social inequalities in diet and improving health and nutrition outcomes include “Bolsa Familia”, a social welfare program in Brazil, and “Oportunidades”, a conditional cash transfer program in Mexico. Even though they did not specifically target inequalities, they approached underlying drivers of social inequalities which often lead to the reduction of dietary inequalities [22].

### Study limitations

Our study has some limitations. First, most interventions were conducted in the Americas and the Caribbean, and implementation of interventions promoting healthy eating might differ in other countries, especially in low income countries. However, we believe that our main conclusion is applicable to all low- and middle-income countries similar to the ones where the studies were conducted, although no intervention is effective in all settings or considered as “one-size fits all”. Second, two interventions targeted several settings in communities (i.e. neighborhoods, schools, and workplaces) [35, 37]. Thus, we could not separate the results obtained in one setting from the others. However, none of these interventions had an impact on social inequalities in diet. Third, due to the small number of studies reporting results by SES, only seven studies could be included in our review, and further research on the effect of interventions on social inequalities in dietary intake in LMICs is required. Fourth, three of the interventions were quasi-experimental [32, 36, 37] so causality between the intervention and the behavioral change can be inferred. Fifth, no study assessed long term dietary behavior change and the reduction of social inequalities in diet and further research is suggested.

## Conclusion

We conclude that in LMICs, agentic interventions to promote healthy eating such as nutrition education are effective to reduce social inequalities in diet when specifically targeting disadvantaged populations. Further research should assess the impact on social inequalities in diet of a combination of agentic and structural approaches in interventions promoting healthy eating.

## Additional files

Additional file 1: Table S1. Free search terms included in the search strategy. (DOC 27 kb)
Additional file 2: Figure S1 Flow diagram of excluded studies. (DOC 63 kb)

## Abbreviations

HICs: High-income countries
LMICs: Low- and middle-income countries
NCDs: Non-communicable diseases
SDH: Social determinants of health
SES: Socioeconomic status
