# Supplementary material for: Interventions promoting healthy eating as a tool for reducing social inequalities in diet in low- and middle-income countries: a systematic review
Source: Int J Equity Health. 2016 Dec 22;15:205. doi: 10.1186/s12939-016-0489-3 (PMC5180409; doi:10.1186/s12939-016-0489-3)
Supplement: Additional file 1: Table S1. — Free search terms included in the search strategy. (DOC 27 kb) [file 12939_2016_489_MOESM1_ESM.doc]

**Additional file 1: Table S1.** Free search terms included in the search strategy.

| Database | Seach terms |
| --- | --- |
| Pubmed | (Diet/nutrition/food AND "socioeconomic status"/"education"/"educational"/"income"/"job position"/"occupation"/"wealth"/"socioeconomic differences"/"social differences"/"social inequalities"/"social disparities") AND "intervention" AND/OR (non communicable diseases OR NCD) AND (developing countries OR low middle income countries) NOT undernutrition NOT children NOT adolescents |
| Scielo | Dieta/nutricion/alimentacion AND intervencion AND socioeconomico/educacion/ingreso |
| Google Scholar | Diet/nutrition/food AND "socioeconomic" /"education"/"income"/"job position"/"occupation"/"wealth"/"socioeconomic differences"/"social differences"/"social inequalities"/"social disparities" AND intitle:intervention AND "non communicable diseases" AND "developing" countries OR low middle income countries |
