# Supplementary material for: Interventions promoting healthy eating as a tool for reducing social inequalities in diet in low- and middle-income countries: a systematic review
Source: Int J Equity Health. 2016 Dec 22;15:205. doi: 10.1186/s12939-016-0489-3 (PMC5180409; doi:10.1186/s12939-016-0489-3)
Supplement: Additional file 2: Figure S1 — Flow diagram of excluded studies. (DOC 63 kb) [file 12939_2016_489_MOESM2_ESM.doc]

**Additional file 2: Figure S1.** Flow diagram of excluded studies.

Databases search

n= 249

Children or adolescents

n=46

High income countries

n=27

No healthy eating outcome

n=15

No intervention assessed or prevalence studies

n=36

No SES assessed or results of whole social strata not shown by SES

n=24

Articles meeting criteria

n= 7

Non peer-reviewed

n=11

Non representative studies

n=15

Only abstract

n=11

Qualitative studies

n=2

Diseased individuals n=41

Diseased individuals or using medications

n=10

Focused on undernutrition

n=4
